# Epigenome-wide association study of asthma and wheeze characterizes loci within *HK1*

**DOI:** 10.1186/s13223-019-0356-z

**Published:** 2019-07-24

**Authors:** Todd M. Everson, Hongmei Zhang, Gabrielle A. Lockett, Akhilesh Kaushal, Melinda Forthofer, Susan L. Ewart, Kimberley Burrows, Caroline L. Relton, Gemma C. Sharp, A. John Henderson, Veeresh K. Patil, Faisal I. Rezwan, S. Hasan Arshad, John W. Holloway, Wilfried Karmaus

**Affiliations:** 1 Department of Epidemiology and Biostatistics, Arnold School of Public Health, University of South Carolina, 915 Greene Street, Columbia, SC 29208 USA; 2 Present Address: Department of Environmental Health, Rollins School of Public Health, Emory University, 1518 Clifton Rd, Atlanta, GA 30322 USA; 3 Division of Epidemiology, Biostatistics and Environmental Health, School of Public Health, University of Memphis, 236A Robison Hall, Memphis, TN 38152 USA; 4 Human Development and Health, Faculty of Medicine, University of Southampton, Southampton General Hospital, Southampton, SO16 6YD UK; 5 Center for Precision and Environmental Health, Baylor College of Medicine, 1 Baylor Plaza, Houston, TX 77030 USA; 6 Present Address: Department of Public Health Sciences at the College of Health and Human Services, University of North Carolina Charlotte, 9201 University City Boulevard, Charlotte, NC 28223 USA; 7 Department of Large Animal Clinical Sciences, Michigan State University, East Lansing, MI USA; 8 MRC Integrative Epidemiology Unit, Population Health Sciences, Bristol Medical School, University of Bristol, Bristol, BS8 2BN UK; 9 Avon Longitudinal Study of Parents and Children, Population Health Sciences, Bristol Medical School, University of Bristol, Bristol, BS8 2BN UK; 10 Clinical and Experimental Sciences Faculty of Medicine, University of Southampton, Southampton General Hospital, Southampton, SO16 6YD UK; 11 The David Hide Asthma and Allergy Research Centre, St Mary’s Hospital, Newport, Isle of Wight UK; 12 NIHR Respiratory Biomedical Research Unit, University Hospital Southampton, Southampton, UK

**Keywords:** ALSPAC, ARIES, Asthma, Expression, *Hexokinase*-*1*, *HK1*, Infant wheeze, Isle of Wight, Methylation

## Abstract

**Background:**

To identify novel epigenetic markers of adolescent asthma and replicate findings in an independent cohort, then explore whether such markers are detectable at birth, predictive of early-life wheeze, and associated with gene expression in cord blood.

**Methods:**

We performed epigenome-wide screening with recursive random forest feature selection and internal validation in the IOW birth cohort. We then tested whether we could replicate these findings in the independent cohort ALSPAC and followed-up our top finding with children of the IOW cohort.

**Results:**

We identified 10 CpG sites associated with adolescent asthma at a 5% false discovery rate (IOW, n = 370), five of which exhibited evidence of associations in the replication study (ALSPAC, n = 720). One site, cg16658191, within *HK1* displayed particularly strong associations after cellular heterogeneity adjustments in both cohorts (OR_IOW_ = 0.17, 95% CI 0.04–0.57) (OR_ALSPAC_ = 0.57, 95% CI 0.38–0.87). Additionally, higher expression of *HK1* (OR = 3.81, 95% CI 1.41–11.77) in cord blood was predictive of wheezing in infancy (n = 82).

**Conclusion:**

We identified novel associations between asthma and wheeze with methylation at cg16658191 and the expression of *HK1*, which may serve as markers of, predictors of, and potentially etiologic factors involved in asthma and early life wheeze.

**Electronic supplementary material:**

The online version of this article (10.1186/s13223-019-0356-z) contains supplementary material, which is available to authorized users.

## Background

Asthma is a common chronic respiratory disease particularly among children [1], causing substantial health care costs [2]. Asthma has complex pathophysiology [3], phenotypic variability [4], and is polygenic with a high heritable component, yet GWAS discoveries can only explain a small fraction of asthma variance [5]. Epigenetic mechanisms, which regulate gene expression potential [6], have received attention in studies of asthma because these mechanisms may be influenced via environmental exposures, particularly exposures that occur in utero [7, 8]. One of the most studied epigenetic mechanism is DNA methylation (DNAm), which is the covalent addition of a methyl group to the DNA at a cytosine residue that is followed by a guanine (CpG site); acting as an important regulator of gene transcription [6].

Recent epigenetic epidemiology studies have associated DNAm in blood with current asthma and/or wheeze in childhood and adolescence [9, 10], in cord blood with childhood asthma [11], and as a possible mediator between maternal and offspring asthma [12]. DNA methylation has also been implicated as a possible mediator of the relationships between both environmental and genetic factors with asthma. For instance, environmental exposure to air pollution is a risk factor for asthma exacerbations as well as asthma onset [13]. Recent studies have shown that air pollution is associated with differential DNAm of *TET1* [14] and *FOXP3* [15], and that differential methylation of these genes associates with asthma, suggesting that epigenetic regulation has a potential mediating role. Additionally, *GSDMB* and *ORMDL3* are two well-recognized asthma susceptibility genes [16], and recent studies have shown that DNAm may be a mediator between genetic variation and the expression of these genes [17]. These studies provide supportive evidence that epigenetic mechanisms may be involved in the etiology of asthma, potentially as intermediates between recognized risk factors and the development of symptoms. A recent epigenome-wide meta-analysis of multiple European cohorts identified robust associations between asthma and blood DNA methylation throughout childhood (4–8 years of age), which retained strong associations with asthma status among isolated eosinophils and these epigenetic signatures were indicative of eosinophil and cytotoxic cell activation [18]. The above studies highlight the mounting evidence that differential epigenetic regulation of specific genes contributes to asthma etiology, and that epigenome-wide approaches have led to the identification of novel asthma-associated epigenomic loci. Performing additional EWAS in independent populations with different ages and different asthmatic phenotypes can improve our understanding of which loci are informative across multiple populations, how these epigenetic variations relate to asthma throughout the life course, and whether their methylation levels correlate with specific phenotypic characteristics, such as inflammation or lung function.

In the current study, we performed an EWAS using both a standard CpG-by-CpG approach as well as an innovative feature selection method to identify novel epigenetic markers of prevalent asthma in 18 years olds, investigated if the identified loci were predictive of early-life wheeze, and whether DNA methylation at these loci were related to gene expression. We first conducted an exploratory epigenome-wide screening study of DNAm in whole blood within the Isle of Wight (IOW) birth cohort, followed by a replication study within the Avon Longitudinal Study of Parents and Children (ALSPAC). Then, with data from the offspring of the IOW birth cohort participants, we tested whether the same associations exist between cord blood DNAm and wheeze without upper respiratory viral infection (cold) within the 1st year of life, followed by testing for associations between gene expression with DNAm and with infant wheeze.

## Methods

### The Isle of Wight birth cohort

The Isle of Wight (IOW) birth cohort is an unselected birth cohort of children born between January 1, 1989 and February 28, 1990 in Isle of Wight, UK. Details about the birth cohort have been described in detail elsewhere [19]. After exclusion of adoptions and prenatal deaths, 1456 children were enrolled and followed-up through to 18 years of age (n = 1313; 90.2% retention). At each follow-up, participants were evaluated for manifestations of allergic disease and administered detailed questionnaires, including study specific questions, as well as questions derived from the International Study of Asthma and Allergies in Childhood (ISAAC), the most extensive international study of asthma, which lead to the development and validation of questions about asthma and wheeze symptoms [20]. Ethical approval was obtained from National Research Ethics Service, NRES Committee South Central—Southampton B for the 18-year follow-up (06/Q1701/34) and NRES Committee South Central—Hampshire B (09/H0504/129) for the follow-up of IOW participants’ offspring; written informed consent was provided by the infants’ parents.

At the 18-year follow-up a subset of participants (n = 370) were selected to take part in an epigenetic screening; this sample is referred to as the ‘IOW F1 sample’ herein. The primary outcome for this study was current asthma defined as having an asthma diagnosis and self-reported wheeze and/or use of asthma medications in the previous 12 months. Those attending the 18 years follow-up in person also performed spirometry and fractional-exhaled nitric oxide (FeNO), which were in accordance with American Thoracic Society (ATS) guidelines [21, 22], as well as allergen sensitization via skin prick tests (SPTs). Lung function assessments were performed using a Koko Spirometer and software with a desktop portable device (PDS Instrumentation, Louisville, USA). FeNO measurements (Niox mino, Aerocrine AB, Solna, Sweden) were obtained prior to spirometric assessments. Atopy was defined as having at least one positive SPT among 11 allergens (cows’ milk, hens’ egg, peanut, cod, house dust mite, cat, dog, *Alternaria alternata*, *Cladosporium herbarium*, grass pollen mix, and tree pollen mix). DNA was extracted from peripheral blood collected at the 18-year follow-up using a salting out procedure.

The IOW offspring (IOW F2 sample) are being enrolled in the IOW 3rd Generation study through ongoing recruitment since 2010. To date, 390 newborns have been enrolled; cord blood samples were collected at birth and have been processed on 111 newborns for DNAm and 82 newborns for gene expression. Questionnaires about allergy and wheeze symptoms were administered to the parents at follow-up visits 3, 6, and 12 months after birth. The primary dependent variable for this sample was parent reported wheeze occurring when the infant had no symptoms of a cold. We also investigated any reported wheeze as an alternate outcome.

### The ALSPAC cohort

The Avon Longitudinal Study of Parents and Children (ALSPAC) is a large, prospective cohort study based in the South West of England. In total, 14,541 pregnant women resident in Avon, UK with expected delivery dates between 1st April 1991 and 31st December 1992 were initially enrolled; 13,988 children were alive at 1 year [23, 24]. Ethical approval for the study was obtained from the ALSPAC Ethics and Law Committee and the Local Research Ethics Committees; written informed consent was provided by all participants. Self-completed questionnaires were administered during pregnancy and then at regular intervals. Current asthma status was obtained around the age of 17 years, defined as a reported doctor’s diagnosis of asthma in addition to reported wheezing, asthma or the use of asthma medication in the previous 12 months. 5036 adolescents had complete phenotype data of which, 720 also had DNA methylation data from whole blood collected at an average age of 17 years. Genome-wide methylation measurements were conducted at the University of Bristol as part of Accessible Resource for Integrated Epigenomic Studies (ARIES) project (http://www.ariesepigenomics.org.uk) [25]. For the purposes of this study, multiple births and children of non-white ethnicity were excluded due to small numbers.

### DNA methylation arrays

In the IOW F1 sample (18 years of age), IOW F2 sample (cord blood), and ALSPAC sample (17 years of age) DNAm was assessed genome-wide using the Illumina Infinium^®^ HumanMethylation450k BeadChip (Illumina, Inc., CA, USA). The details of data processing steps are provided in Additional file 1: Method S1. Briefly, quality control and preprocessing methods for both cohorts included background correction, probe-type standardization, batch effect adjustments, and exclusion of potentially problematic probes. Methylation levels were calculated as beta (β) values, which can be interpreted as percent methylation. Because β-values can suffer from severe heteroscedasticity, M-values were calculated via log_2_(β/(1 − β)) which better approximate a normal distribution. Cellular heterogeneity of blood samples was assessed by estimating the proportions of CD8^+^ T-cells, CD4^+^ T-cells, natural killer cells, B-cells, monocytes, eosinophils and other granulocytes [26, 27] via the estimateCellCounts function in R. These proportions were included in our regression models as potential confounders of the relationship between DNA methylation and current asthma.

### Gene expression array

At birth, IOW F2 cord blood samples were collected into PAXgene Bone Marrow RNA Tubes and RNA extracted using PAXgene RNA kits (PreAnalytiX GmbH, Switzerland). RNA integrity was verified with the Agilent 2100 Bioanalyzer system. Genome-wide mRNA expression was assessed via one color (Cy3) experiments with the Agilent (Agilent Technologies, Santa Clara, CA) SurePrint G3 Human Gene Expression 8 × 60 k v2 microarray kits. Array content was sourced from RefSeq, Ensembl, UniGene, and GenBank databases and provides full coverage of the human transcriptome in 50,599 biological features (including replicate probes and control probes). The oligos were 60mer in length and each transcript was tagged at least once and some had multiple tagging oligos for genes with documented splice variants. Data QC indices and analyses were performed with Agilent GeneSpring software. These data were then percent shift normalized and log_2_-transformed.

### Statistical analyses (discovery—IOW F1)

We randomly divided the IOW F1 (18 years of age) sample into two independent sub-samples. The stage-1 data (n_S1_ = 91) were used for random forest (RF) feature selection because RFs rely on few statistical assumptions, are efficient with high-dimensional data, are robust to outliers and noise, and produce measures of variable importance [28, 29]. This feature selection technique was utilized in a recent epigenetic study of atopy that yielded many replicable loci [30]. The RF algorithm has a tendency to produce a predictor that is overfit to the supplied data; however, in our study RF was applied to select features based on variable importance rather than prediction. In addition, the RF algorithm was only applied to a subset of the IOW data to further diminish the possibility of overfitting, allowing us to examine the associations between DNAm and asthma in a statistically independent dataset. The stage-2 sample (n_S2_ = 279) was larger to retain greater power, which was necessary for hypothesis testing and multiple testing adjustment.

Recursive RF feature selection was implemented on the stage-1 sample (n_s1_ = 91) to select the CpGs most informative for asthma. We utilized balanced sampling, tested 10% of predictors per node (*mtry* = 0.10) and grew forests with 7500 trees (*ntree* = 7500). We implemented the RF recursively: (1) ran the RF algorithm on all available predictors (248,336 CpGs) via the randomForest package in R, (2) extracted out-of-bag (OOB) misclassification rates and variable importance measures (VIMs), (3) sorted the predictors by their VIMs, (4) excluded half of the predictors with the smallest VIMs, and (5) repeated the sequence until the asthma-specific misclassification levelled off. Predictors from the final iteration were selected for stage-2 analyses.

M-values for the selected CpGs were tested for their associations with asthma status with logistic regression after trimming potential strong outliers identified with adjusted boxplots. We generated false discovery rate (FDR) adjusted p-values [31] via the q-value package in R; CpGs within a 5% FDR (q-values < 0.05) were considered ‘discovered’ and were candidates for the replication study.

Finally, we also performed a traditional EWAS regressing the beta-values for each individual loci on asthma status in unadjusted linear models, and models adjusted for sex, CD4^+^ T-cells, CD8^+^ T-cells, monocytes, eosinophils, natural killer, and granulocytes. Models that produced association within a 5% FDR (q-values < 0.05) were considered statistically significant.

### Statistical analyses (independent replication—ALSPAC)

Candidate CpGs were tested for their associations with asthma in the independent cohort, ALSPAC (N = 720). To assess consistency of associations, results from the ALSPAC cohort were compared to results from the full IOW F1 sample (N = 370) using two logistic regression models for each CpG site: a crude model between M-values and asthma, and a second model adjusting for sex and estimated cell-type proportions of CD8^+^ T-cells, CD4^+^ T-cells, natural killer cells, B-cells, monocytes, eosinophils and other granulocytes, which were estimated from the methylation array data [26, 27]. ALSPAC also included batch variables (Additional file 1: Method S2), to adjust for technical variations across the DNAm arrays. Statistical significance was determined at α of 0.05.

### Statistical analyses (functional validation—IOW F2)

Among the successfully replicated loci, wheeze without cold and any wheeze were modeled with logistic regression. This included all newborns for which at least one infant follow-up visit had been completed (n = 111 for DNAm models and n = 82 for expression models). Cord blood proportions of CD8^+^ T-cells, CD4^+^ T-cells, natural killer cells, B-cells, monocytes, granulocytes, and nucleated red blood cells (nRBCs) were estimated via the estimateCellCounts function [26] using a cord blood reference panel [32]. We adjusted for season of birth, infant sex, and cell-type proportions. Statistical significance was determined at α of 0.05.

## Results

### Sample characteristics and study flow chart

**Fig. 1:**
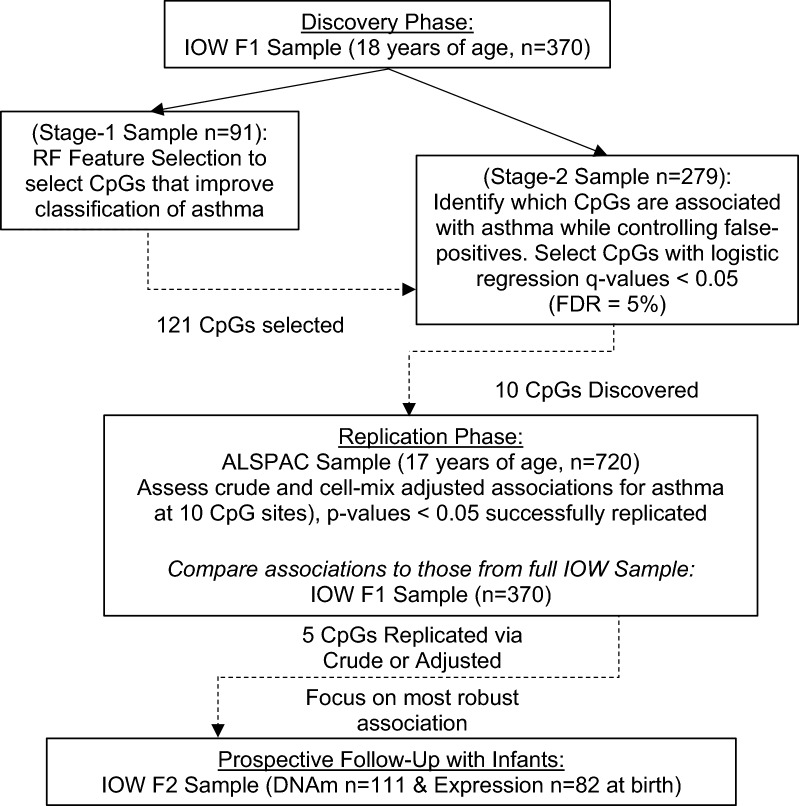
Flowchart of analyses and results for each stage of the study. *IOW* Isle of Wight, *F1* first generation sample, *F2* second generation sample, *ALSPAC* Avon Longitudinal Study of Parents and Children, *DNAm* DNA methylation

A flowchart of all analyses is provided in Fig. 1. The subjects in the IOW F1 discovery sample were all 18 years old, predominantly female (66.2%) and 13.9% (n = 51) of participants were asthmatic. Asthmatics were more likely to be atopic (66.0% vs 29.5%), have lower FEV_1_/FVC Ratio (means: 0.83 vs. 0.88), greater FeNO (medians: 21.0 vs 14.0) and have higher proportions of B-cells (0.046 vs 0.039) and eosinophils (0.045 vs 0.021) (Table 1). The average age of subjects in the ALSPAC sample was 17 years old; 16.7% of the ALSPAC sample had asthma and 56.3% of participants were female. The IOW stage-1 and stage-2 samples, utilized for feature selection and internal validation respectively, had similar distributions of all covariates (Additional file 2: Table S1).

**Table 1:** Baseline characteristics among those with and without asthma in the IOW F1 sample (18 years of age)

Categorical factors	Asthmatics (n = 51)		Non-asthmatics (n = 319)	
	n	Percent	n	Percent
Female sex	35	68.6	210	65.8
Atopy	33	66.0	93	29.5

Continuous factors	n	Mean (SD)	n	Mean (SD)
FVC	49	4.50 (0.874)	307	4.48 (0.857)
FEV_1_	49	3.74 (0.750)	307	3.94 (0.719)
FEV_1_/FVC Ratio	49	0.83 (0.088)	307	0.88 (0.064)
FeNO^a^	47	21.00 (60.00)	300	14.0 (10.50)

Estimated cell proportions	n	Mean (SD)	n	Mean (SD)
CD8^+^ T-Cells	51	0.101 (0.082)	319	0.098 (0.054)
CD4^+^ T-Cells	51	0.121 (0.052)	319	0.110 (0.047)
Natural Killer Cells	51	0.055 (0.061)	319	0.070 (0.062)
B-Cells	51	0.046 (0.023)	319	0.039 (0.024)
Monocytes	51	0.072 (0.024)	319	0.074 (0.024)
Eosinophils	51	0.045 (0.045)	319	0.021 (0.025)
Neutrophils	51	0.558 (0.118)	319	0.589 (0.114)

*FEV*_*1*_ forced expiratory volume in 1 s, *FVC* forced vital capacity, *FeNO* fractional-exhaled nitric oxide

^a^Due to heavy positive skew of FeNO, medians (interquartile ranges) are presented here

### Discovery phase (stage-1 feature selection)

Recursive RF feature selection was implemented on the stage-1 sub-sample (n_s1_ = 91), with a starting set of 248,336 CpG sites. The asthma-specific misclassification rates levelled off at the 12th iteration of the recursive RF algorithm, meaning that further reductions in the number of features would result in loss of information about asthma-associated loci. Thus, the 121 features (CpG sites) included in the 12th iteration (Additional file 3: Figure S1) were selected for stage-2 analysis.

### Discovery phase (stage-2 logistic regression)

The stage-2 analysis was performed in an independent sub-sample (n_s2_ = 279), to test the associations between DNA methylation and asthma at the 121 selected CpG sites with logistic regression. Of the 121 CpGs, 10 were associated with asthma at a 5% FDR (q-values < 0.05) (Additional file 2: Table S2). For all 10 sites, lower methylation was associated with greater odds of asthma. Adjustment for cellular heterogeneity substantially attenuated many of the parameter estimates and none of the adjusted models retained 5% FDR-significant q-values. However, the parameter estimates for the top five hits were mostly unperturbed and retained at least nominally significant p-values (< 0.05); in the case of cg16658191 and cg25578728, the magnitude of the associations became stronger after cell-mixture adjustment.

### Replication analysis in ALSPAC

We then aimed to see whether the associations observed in IOW could be replicated in an independent cohort, ALSPAC. To compare associations between the IOW and ALSPAC cohorts, we produced odds ratios (ORs) and 95% confidence intervals (CIs) using the pooled IOW samples from stage-1 and stage-2 (IOW F1 n = 370) (Table 2) and ALSPAC (n = 720) (Table 3) for the 10 FDR-significant CpG sites. All 10 CpGs exhibited the same direction of association, while 3 of these associations were statistically significant after cell-mix adjustment (p-values < 0.05) (Table 3; Additional file 2: Table S3): cg04359558 (*LITAF*), cg13753183 (*APTX*), and cg16658191 (*HK1*). These CpGs have been annotated with genomic information and function (Table 4). Differences in the distributions of cell-types are presented in Additional file 2: Table S4).

**Table 2:** Crude and adjusted associations between M-values and asthma in the IOW F1 (18 years of age, n = 370) sample via logistic regression

CpG annotations		DNAm distribution		Crude model		Adjusted model^a^	
CpG ID	Gene	Median	(5th, 95th pct.)	OR	CI	OR	CI
cg00100703	*UNC45B*	0.93	(0.89, 0.95)	0.12	(0.05, 0.28)	0.18	(0.06, 0.50)
cg01069468	*SYNGAP1*	0.88	(0.84, 0.91)	0.12	(0.04, 0.31)	0.39	(0.08, 1.72)
cg04359558	*LITAF*	0.93	(0.87, 0.96)	0.23	(0.12, 0.42)	0.34	(0.17, 0.68)
cg06866208	–	0.81	(0.73, 0.84)	0.14	(0.05, 0.34)	0.40	(0.09, 1.72)
cg07948085	–	0.89	(0.84, 0.93)	0.16	(0.07, 0.34)	0.26	(0.09, 0.74)
cg09241885	*C20orf118*	0.89	(0.80, 0.93)	0.29	(0.16, 0.49)	0.41	(0.17, 0.99)
cg11310939	*MARCH3*	0.91	(0.86, 0.92)	0.19	(0.09, 0.40)	0.45	(0.15, 1.44)
cg13753183	*APTX*	0.90	(0.86, 0.93)	0.11	(0.04, 0.30)	0.30	(0.06, 1.38)
cg16658191	*HK1*	0.93	(0.89, 0.95)	0.12	(0.05, 0.27)	0.17	(0.04, 0.57)
cg25578728	*CHD7*	0.90	(0.85, 0.94)	0.24	(0.11, 0.46)	0.27	(0.11, 0.60)

*OR* odds ratio, *CI* 95% confidence intervals, *IOW* Isle of Wight, *pct.* percentile

^a^Adjusted for cell mixture and sex

**Table 3:** Replication of crude and adjusted associations between M-values and asthma in the ALSPAC (17 years of age, n = 720) sample via logistic regression

CpG annotations		Crude model		Adjusted model^a^	
CpG ID	Gene	OR	CI	OR	CI
cg00100703	*UNC45B*	0.71	(0.52, 0.96)	0.77	(0.52, 1.15)
cg01046943	*NUP210*	0.93	(0.71, 1.20)	0.83	(0.62, 1.11)
cg04359558	*LITAF*	0.75	(0.56, 1.00)	0.61	(0.40, 0.94)
cg06866208	–	1.01	(0.70, 1.44)	0.86	(0.38, 1.97)
cg07948085	–	0.63	(0.43, 0.91)	0.70	(0.45, 1.06)
cg09241885	*C20orf118*	0.85	(0.64, 1.13)	0.75	(0.51, 1.10)
cg11310939	*MARCH3*	0.78	(0.60, 1.02)	0.90	(0.65, 1.24)
cg13753183	*APTX*	0.76	(0.58, 1.00)	0.67	(0.46, 0.98)
cg16658191	*HK1*	0.51	(0.37, 0.72)	0.57	(0.38, 0.87)
cg25578728	*CHD7*	0.73	(0.53, 1.02)	0.78	(0.50, 1.24)

*OR* odds ratio, *CI* 95% confidence intervals, *ALSPAC* Avon Longitudinal Study of Parents and Children, *pct.* percentile

^a^Adjusted for cell mixture, sex, and batch variables

**Table 4:** Annotations and biological functions of genes associated with CpG sites associated with asthma in the replication study via either the adjusted or unadjusted models

CpG ID	Region	Gene ID	Gene name	Function
cg04359558	Body	*LITAF*	*Lipopolysaccharide*-*Induced TNF*-*α Factor*	DNA binding-protein that promotes inflammatory cytokine expression; involved in apoptotic signaling and inhibition of proliferation [46, 47]
cg13753183	Body; 1st Exon; 5′UTR	*APTX*	*Aprataxin*	Involved in DNA repair; mutations have been associated with ataxia-ocular apraxia [49]
cg16658191	Body; 1st Exon	*HK1*	*Hexokinase*-*1*	Involved in glucose metabolism [34] and inhibition of apoptotic signaling [40]

*Ch* Chromosome, *UTR* untranslated region

Adjusting for estimated cell mixtures attenuated most ORs, and led to some discordance between IOW and ALSPAC, with only cg04359558 and cg16658191 exhibiting significant associations with asthma in both cohorts after cell-type adjustments. Only our top-hit (cg16658191) was significantly associated with asthma in all models across both cohorts.

Some of the tested CpGs were observed to have moderate-to-strong Spearman correlations (cg06866208, cg07948085, cg09241885, cg11310939, cg13753183, cg16658191) with the proportions of estimated eosinophils (range of rho values: − 0.51 to − 0.59, p-values < 0.0001) and were also moderately correlated with each other (range of rho values: 0.26 to 0.49, p-values < 0.0001) (Additional file 3: Figure S2), suggesting that methylation levels at these CpGs may be partial markers of eosinophils.

Given the inconsistent confounding effects of cell-type, we considered cg16658191 within the *hexokinase*-*1* (*HK1*) gene as the finding with the most consistent evidence for an association with asthma and carried this CpG forward for cross-sectional analyses with allergy, inflammation and lung-function, as well as prospective analyses with infant respiratory outcomes.

### HK1 DNA methylation is associated with allergy, inflammation and lung function

**Fig. 2:**
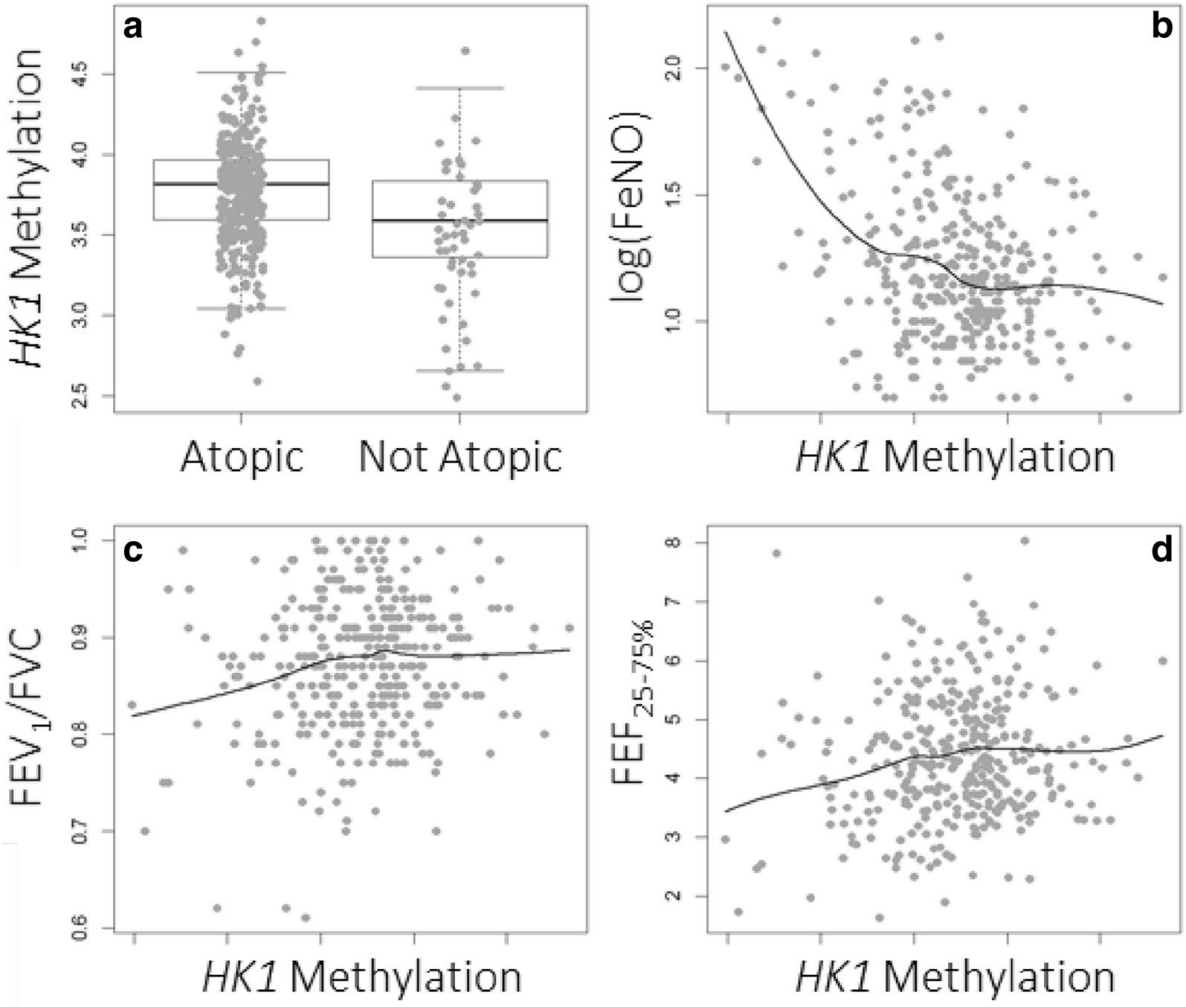
Variation in DNAm (beta-values) at cg16658191 by **a** adolescent atopy, **b** log(FeNO), **c** FEV_1_/FVC, and **d** FEF_25–75%_, within the IOW F1 sample. *HK1*, hexokinase-1; FeNO, fractional exhaled nitric oxide; FEV_1_/FVC, forced expiratory volume in one second divided by the forced vital capacity; FEF_25–75%_, forced expiratory flow at 25–75% of forced vital capacity

We found that DNAm at cg16658191 was lower among those with atopy (T-test: *HK1* p-value < 0.001) and had an inverse non-linear association with logFeNO (rho = − 0.22, p-value < 0.0001), suggesting that it is involved in allergic sensitization and airway inflammation. Additionally, those with lower DNAm at this locus tended to have lower FEV_1_/FVC (rho = 0.10, p-value = 0.057) and FEF_25–75%_ (rho = 0.095, p-value = 0.075) though these correlations were not statistically significant (Fig. 2).

### Prospective follow-up for HK1 associations in infants

We then performed follow-up analyses for associations of our top locus, cg16658191, with wheeze during infancy and variations in gene-expression in the IOW F2 sample. Infants with lower levels of cord blood DNAm at cg16658191 had greater odds of wheeze without cold within the 1st year of life (Table 5), though adjustments for estimated cell-types confounded this association, particularly due to a strong correlation with nRBCs (rho = − 0.84, p-value < 0.0001) and moderate correlation with granulocytes (rho = 0.59, p-value < 0.0001). Additionally, DNAm at cg16658191 was inversely associated with the expression of *HK1* (rho = − 0.22, p-value = 0.039) and increased expression of *HK1* was associated with increased odds of wheeze without cold and odds of any wheeze, during the 1st year of life. Interestingly, these associations became stronger after adjusting for cellular heterogeneity (Table 5).

**Table 5:** Associations between cord blood DNAm at cg16658191 and expression of *HK1* with infant wheeze without cold and any wheeze within the IOW F2 sample

Exposure	Outcomes					
	Wheeze without cough/cold			Any wheeze		
	OR	p-value	95% CI	OR	p-value	95% CI
cg16658191 (n = 111)						
Crude model	0.23	0.0097	(0.07–0.68)	0.58	0.23	(0.23–1.41)
Adjusted model 1^a^	0.21	0.0062	(0.06–0.61)	0.57	0.23	(0.22–1.40)
Adjusted model 2^b^	0.37	0.30	(0.05–2.35)	2.42	0.27	(0.52–12.18)
*HK1* (A_23_P217958) (n = 82)						
Crude model	3.14	0.0183	(1.26–8.62)	3.44	0.0045	(1.54–8.59)
Adjusted model 1^a^	3.62	0.015	(1.34–10.95)	3.22	0.0075	(1.42–8.08)
Adjusted model 2^b^	7.51	0.0085	(1.89–40.50)	4.89	0.0087	(1.63–18.15)

OR, Odds Ratio; CI, 95% confidence interval; CB, cord blood; *HK1*, hexokinase-1; w/o, without

^a^Adjusted for infant sex and season of birth

^b^Adjusted for infant sex, season of birth, and cell mixture

### Discovery of epigenomic loci associated with asthma (traditional EWAS approach)

Finally, we examined DNAm-asthma associations using a standard EWAS approach, regressing methylation levels for all CpGs on asthma status in unadjusted models and models adjusted for sex, CD4^+^ T-cells, CD8^+^ T-cells, monocytes, eosinophils, natural killer, and granulocytes. In the unadjusted models, 148 CpGs were significantly associated (FDR 5%) with asthma status. However, adjusting for sex and cell mixture resulted in attenuation of most of these results and none of the adjusted models produced FDR-significant associations. We compared the results from our models, unadjusted (Additional file 2: Table S5) and adjusted (Additional file 2: Table S6) that yielded p-values < 0.001, to the results from a prior EWAS in ALSPAC for current asthma and current wheeze at ages 7.5 and 16.5 years that yielded p-values < 0.001 [10]. Of the 674 CpGs that were associated with asthma (p-value < 0.001) in IOW prior to cell-type adjustment, 20 CpGs yielded p-values < 0.001 for all four models in ALSPAC (Additional file 2: Table S7). However, only one CpG yielded a p-value < 0.001 in the IOW and a p-value < 0.001 in ALSPAC when adjusting for cell mixture, and that was cg16658191. We also compared our results to the asthma-associated CpGs identified in a meta-analysis of children between the ages 4 and 8 years old [18] at the 11 (out of 14) sites that passed QC in our study. Although all 11 sites yielded nominally significant inverse associations with asthma in IOW (Additional file 2: Table S8) only cg10142874 retained even a nominal association with asthma in IOW after cell-mix adjustment (p-value = 0.013).

## Discussion

We performed an epigenome-wide association study of current asthma in the IOW cohort utilizing two statistical approaches and a replication analysis in an independent population. We identified that lower DNAm at cg16658191 within the 1st exon of *HK1* as a marker of current asthma. This CpG was identified via random forest feature selection and confirmed using standard EWAS, and was replicated within an independent cohort (ALSPAC). We then produced similar associations between DNAm of cg16658191 and the expression of *HK1* in cord blood with infant wheeze in the children of the IOW cohort. We also observed functional evidence of *HK1*′s involvement in infant wheeze using gene expression data that exhibited the expected associations with infant wheeze, given that promoter and first exon methylation are most commonly associated with repression of gene expression [6, 33]. DNAm at cg16658191, which is within the body and/or first exon, was inversely associated with *HK1* expression and we showed that higher expression of *HK1* was predictive of wheezing without a cold during infancy. The *HK1* gene resides in 10q22.1 and encodes a protein that is integral in the first step of glycolysis [34] and in apoptotic resistance [35]. The consistency of these associations across different ages, with different respiratory outcomes, and utilizing both DNAm and gene expression as predictors, suggests that this gene may play an important role in the predisposition for wheezing and/or asthma.

We found that many of our RF-identified hits, including cg16658191, were inversely correlated with eosinophil counts in adult blood, similar to what was observed by Arathimos et al. [10]. However, confounding by cell-mixture may not be limited to eosinophil proportions. For instance, because of its crucial role in glucose metabolism, *HK1* is highly expressed by erythrocytes [36]. This is consistent with the strong inverse correlation we observed between cg16658191 and estimated nRBC proportions in cord blood, which could indicate prematurity, restricted growth, or pregnancy complications [37]. Additionally, premature and low birth weight neonates are predisposed to early-life respiratory morbidity [38, 39]. However, adjustments for weeks of gestation did not appreciably alter our results (data not shown).

Though DNAm at cg16658191 may, in part, be a marker of high eosinophil counts in adult blood and nRBCs in cord blood, we found that, in addition to the relationship between DNAm and asthma, *HK1* expression was strongly and significantly associated with infant wheeze even after cell-type adjustments. These findings suggest a role for DNAm of the *HK1* gene in asthma and wheeze etiology that is independent of cell-type proportions, possibly through differential epigenetic regulation within a subset of asthma-associated cell-types. However, it is difficult to disentangle such relationships in studies that utilize tissues composed of mixed cell populations, such as blood. *HK1* is involved in apoptotic resistance via binding to and stabilizing the mitochondrial membrane, whereas the dissociation of *HK1* from the membrane makes those cells more susceptible to apoptosis [40]. Up-regulation of *HK1* resulting in increased apoptotic resistance has been observed in cancerous cells [41] and HIV-1 infected macrophages [42]. Apoptotic-resistant pro-inflammatory cells are known to lead to prolonged inflammation [43] and apoptosis appears to be delayed in neutrophils [44] and T-lymphocytes [45] of asthmatics. This provides a possible mechanism through which *HK1* epigenetic regulation and expression by immune cells may be involved in asthma and wheeze etiology.

We also examined relationships between DNAm and asthma using a more traditional EWAS approach, regressing the methylation beta-values for each loci on asthma status, and compared our findings to two recent EWAS, one performed by ALSPAC [10] and a meta-analysis of childhood asthma from multiple European cohorts [18]. This approach identified 148 CpGs that were significantly associated (5% FDR) with asthma prior to cell-type adjustment, and no FDR-significant findings after adjustment. We also found very little consistency in observed associations between our study and the ALSPAC study after adjusting for cell mixture. Only cg16658191 demonstrated an association with current asthma and wheeze in the fully adjusted models (p < 0.001) in both the IOW and ALSPAC. When comparing our traditional EWAS results to a meta-analysis of childhood asthma, only cg10142874 from that meta-analysis yielded an even nominally-significant association with asthma in IOW after cell-mixture adjustment.

Strengths of our study included the use of multiple samples to discover and replicate our findings, supported by gene expression studies, and the use of a validated tool, the ISAAC core questionnaire, to define current asthma status. However, it is also important to recognize this study’s limitations. One limitation is that detection of CpGs in the IOW birth cohort and the replication study in ALSPAC investigated concurrent associations. Hence, reverse causation in which asthma may result in differential methylation of *HK1* cannot be excluded. Differences between the two cohorts, confounding by cell-mixture, errors in cell-mixture estimates, and asthma heterogeneity may have limited replicability of more loci after additional adjustments. The discovery and replication samples were similar in sex-distribution, prevalence of asthma, and age, but differed in estimated cell-type distributions (Additional file 2: Table S4). Additionally, though the estimated cell-proportions are imperfect, we utilized the gold standard for predicting cell mixtures from DNAm arrays [27] and comprehensively evaluated the impact of cellular heterogeneity on our findings. There is also the possibility of residual confounding, perhaps by genotype. Numerous SNPs have been implicated as asthma susceptibility loci, and some SNPs have been shown to influence the methylation status of CpG sites. We cannot rule out the possibility that our findings are markers of upstream genetic effects on both DNA methylation and asthma susceptibility. It is also important to point out that the relationships we observed between DNAm and expression of *HK1* in cord blood with infant wheeze, cannot be directly extrapolated to asthma. It is unclear whether DNAm patterns of *HK1* in cord blood are informative for the later development of asthma, although our findings provide evidence that lower DNAm and increased expression of *HK1* in cord blood are associated with wheezing in the 1st year of life. Finally, asthma is a heterogeneous condition, in which different phenotypes may arise via different underlying physiological mechanisms [3]. We showed that *HK1* DNAm levels were also strongly associated with atopy and FeNO, which may indicate that the regulation of this gene is particularly important in allergic-asthma. This also raises the possibility that some of our other discovered, but not replicated, loci may be associated with specific asthma-phenotypes. If the prevalences of these phenotypes differ between IOW and ALSPAC, this may have contributed to the discordant results. Interestingly, some of the CpGs with discordant results between the two cohorts were within genes or genomic regions that have previously been associated with asthma or are involved in apoptotic signaling, like *HK1*. For instance, cg04359558 is within the body of *LITAF*, a gene that encodes a DNA-binding protein that promotes the expression of TNF-α and other cytokines known to be involved in pro-inflammatory and apoptotic signaling [46, 47]. *UNC45B*, annotated to cg00100703, lies within the asthma susceptibility region 17q12–21 [48], though this particular gene has not previously been linked to asthma.

## Conclusions

In summary, we discovered a novel epigenetic association with adolescent asthma at cg16658191 within *HK1*, whose DNAm and expression levels in cord blood were also associated with infant wheeze without cold. In addition, the association of cg16658191 with asthma was replicated in an independent cohort. However, we also found that our findings may be affected, at least in part, by heterogeneous cell-mixtures. Further research is required to determine whether these observed associations are reproducible in other populations, particularly with different racial and ethnic characteristics, and whether some of these loci are differentially regulated between those with and without asthma in specific cell-type populations such as eosinophils.

## Additional files

**Additional file 1: Method S1.** Cohort-Specific DNA-M preprocessing steps. **Methods S2.** SVA to account for technical variations in ALSPAC.

**Additional file 2:** This file includes supplemental Tables (S1–S8)—**Table S1.** Comparison of cell-proportions and lung function variables across the Stage-1 (n = 91) and Stage-2 (n = 279) samples from the IOW F1 Sample. **Table S2.** Parameter estimates from logistic regression models performed in the Stage-2 sample (ns2 = 279), regressing current asthma status on DNA methylation M-values for all CpGs selected from the Stage-1 analysis. **Table S3.** Parameter estimates, standard errors, and p-values to compare the IOW F1 results to the ALSPAC replication results for CpGs that yielded an association within a 5% FDR in the Stage-2 analysis. **Table S4.** Comparing adjustment covariates between the IOW F1 sample and the ALSPAC sample; the within cohort comparisons are testing for differences in these variables between those with and without asthma. **Table S5.** Results from linear asthma EWAS in IOW F1 (n = 370), in which methylation beta values were regressed on asthma status, unadjusted for possible confounders. **Table S6.** Results from linear asthma EWAS in IOW F1 (n = 370), in which methylation beta values were regressed on asthmat status, adjusted for sex, CD4T cells, CD8T cells, Monocytes, Natural Killers, Eosinophils, and other Granulocytes. **Table S7.** CpGs that yielded unadjusted association (p < 0.001) with current asthma in IOW F1 (unadjusted models) that were also identified as having unadjusted associations with current asthma and wheeze at age 7.5 and 16.5 years old in the previously published ALSPAC EWAS. **Table S8.** Associations between asthma and DNA methylation within the IOW cohort (unadjusted models) at the 14 CpGs that were identified as being asthma associated in a meta analysis of European children.

**Additional file 3:** This file includes supplemental figures (S1, S2)—**Figure S1.** Tracking of the misclassification rates (y-axis) across iterations (x-axis) of the recursive RF feature selection. **Figure S2.** Correlations (Spearman) between estimated cell-proportions in blood and DNAM M-values for the 10 CpGs that were identified as candidates for the replication study. Statistically significant correlations are designated at p-values < 0.05 (^*^), p-values < 0.001 (^**^), and p-values < 0.001 (^***^).

## Data Availability

The minimal data sets analyzed in the current study are available from the corresponding author upon reasonable request. For access to the full Isle of Wight Cohort data please see: http://www.allergyresearch.org.uk/studies/birth-cohort/- cohort-data-use; for access to ALSPAC data please see: http://www.bristol.ac.uk/alspac/researchers/access/.

## Abbreviations

ALSPAC: Avon Longitudinal Study of Parents and Children
DNAm: DNA methylation
HK1: hexokinase-1
IOW: Isle of Wight
OOB: out of bag
RF: random forest
VIM: variable importance measure
