# Supplementary material for: Epigenome-wide association study of asthma and wheeze characterizes loci within HK1
Source: Allergy Asthma Clin Immunol. 2019 Jul 24;15:43. doi: 10.1186/s13223-019-0356-z (PMC6657035; doi:10.1186/s13223-019-0356-z)
Supplement: Supplementary file 1 — Additional file 1: Method S1. Cohort-Specific DNA-M preprocessing steps. Methods S2. SVA to account for technical variations in ALSPAC. [file 13223_2019_356_MOESM1_ESM.docx]

**Methods S1: Cohort-Specific DNA-M preprocessing steps.**

In the IOW F1 and F2 Samples, DNA concentration was determined by the PicoGreen dsDNA quantitation kit (Molecular Probes, Inc., OR, USA). One microgram of DNA was bisulfite-treated for cytosine to thymine conversion using the EZ 96-DNA methylation kit (Zymo Research, CA, USA), following the manufacturer's standard protocol. Genome-wide DNA methylation was assessed using the Illumina Infinium® HumanMethylation450k BeadChip (Illumina, Inc., CA, USA). The BeadChips were scanned using a BeadStation, and the methylation levels were calculated for each queried CpG locus using the Methylation Module of BeadStudio software. Arrays were processed using a standard protocol as described elsewhere^1^, with multiple identical control samples assigned to each batch to assess assay variability and samples were randomly distributed on microarrays to control against batch effects. The program for data cleaning was written in R (R Development Core Team, 2012); quality control and assurance procedures included probe-type standardization, peak-correction with Bioconductor IMA (Illumina methylation analyzer) package^2^ and batch adjustments via combat. Probes whose detection p-values > 0.01 in >10% of the samples, measured DNA-M at sex-specific (X and Y) CpGs, or with SNPs that had a minor allele frequency (MAF) > 1.0% and within 10 nucleotides of, or directly at, single base extension via dbSNP137^3^ were excluded, resulting in a final set of 248,336 CpGs for analysis.

In ALSPAC, peripheral blood samples were collected according to standard procedures, spun and frozen at -80˚C. DNA was bisulfite converted using Zymo EZ DNA MethylationTM kit (Zymo, Irvine, CA). The Illumina Infinium® HumanMethylation450k BeadChip assay was used to measure genome-wide methylation levels at the University of Bristol as part of Accessible Resource for Integrated Epigenomic Studies (ARIES) project [ariesepigenomics.org.uk]^4^. Please note that the study website contains details of all the data that are available through a fully searchable data dictionary (<http://www.bris.ac.uk/alspac/researchers/data-access/data-dictionary/>). 1,018 mother-offspring pairs in the ALSPAC cohort were selected for the ARIES project based on availability of DNA samples. Of the 1,018 mother-offspring pairs, 974 adolescents had methylation data which successfully passed QC. Arrays were scanned using the Illumina iScan and initial quality review was assessed using GenomeStudio (version 2011.1). Samples were semi-randomly distributed across slides. Samples with >20% probes with a detection p-value ≥ 0.01 failed quality control and were repeated. The methylation data was pre-processed according to the subset quantile normalization approach^5^ using the WateRmelon package in R (version 3.0.1). All CpGs requested for the replication study were present in the ALSPAC sample.

1. Bibikova M, Barnes B, Tsan C, et al. High density DNA methylation array with single CpG site resolution. *Genomics*. 2011;98:288-295. doi:10.1016/j.ygeno.2011.07.007.

2. Wang D, Yan L, Hu Q, et al. IMA: An R package for high-throughput analysis of Illumina’s 450K Infinium methylation data. *Bioinformatics*. 2012.

3. Chen YA, Lemire M, Choufani S, et al. Discovery of cross-reactive probes and polymorphic CpGs in the Illumina Infinium HumanMethylation450 microarray. *Epigenetics*. 2013;8(2):203-209. doi:10.4161/epi.23470.

4. Relton CL, Gaunt T, McArdle W, et al. Data Resource Profile: Accessible Resource for Integrated Epigenomic Studies (ARIES). *Int J Epidemiol*. 2015:1181-1190. doi:10.1093/ije/dyv072.

5. Touleimat N, Tost J. Complete pipeline for Infinium® Human Methylation 450K BeadChip data processing using subset quantile normalization for accurate DNA methylation estimation. *Epigenomics*. 2012;4(3):325-341. doi:10.2217/EPI.12.21.

**Methods S2: SVA to account for technical variations in ALSPAC.**

The purpose-built laboratory information management system (LIMS) was used to record a range of batch variables during data generation. In addition, LIMS also reported QC metrics from the standard control probes on the HumanMethylation450k BeadChip for each sample back to the laboratory. Bisulphite conversion batch (96-well plate) was identified as the largest contributor to batch effect from those recorded. Adjustment of batch at the slide level was inappropriate due to the small number of samples for each time-point present on each slide. Previous ALSPAC analysis with methylation data has shown that running models adjusted for bisulfite conversion batch caused non-singular fit errors (due to small batches). Batch was therefore included in all analyses by adding several surrogate variables generated using the sva() function in the SVA R package. Surrogate variables were generated separately for each model. 10 SVs were generated and only those that were not associated with the outcome were included as covariates within each model. SVs are used to remove unwanted and unmeasured sources of bias. By including SV generation of all exposures, the SVs should capture unknown sources of variation and bias whilst maintaining the contribution of variation by the measured covariates and cell types. For this reason cell types was included in the SV analysis irrespective of whether the cell proportions are added as a covariate in that particular model in order to still observe the effect of cell proportions in the relevant models.
