# Supplementary material for: Epigenome-wide association study of asthma and wheeze characterizes loci within HK1
Source: Allergy Asthma Clin Immunol. 2019 Jul 24;15:43. doi: 10.1186/s13223-019-0356-z (PMC6657035; doi:10.1186/s13223-019-0356-z)

**Figure S1:** Tracking of the misclassification rates (y-axis) across iterations (x-axis) of the recursive RF feature selection.


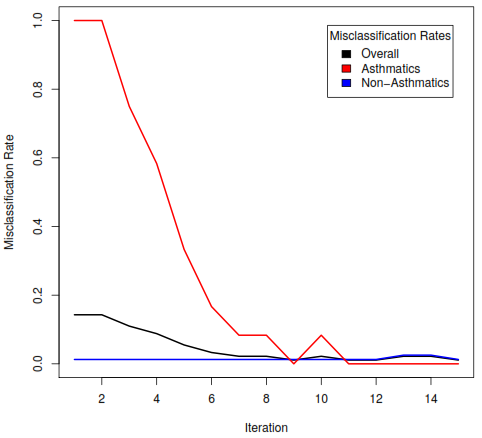


**Figure S2:** Correlations (Spearman) between estimated cell-proportions in blood and DNAM M-values for the 10 CpGs that were identified as candidates for the replication study. Statistically significant correlations are designated at p-values < 0.05 (^*^), p-values < 0.001 (^**^), and p-values < 0.001 (^***^).


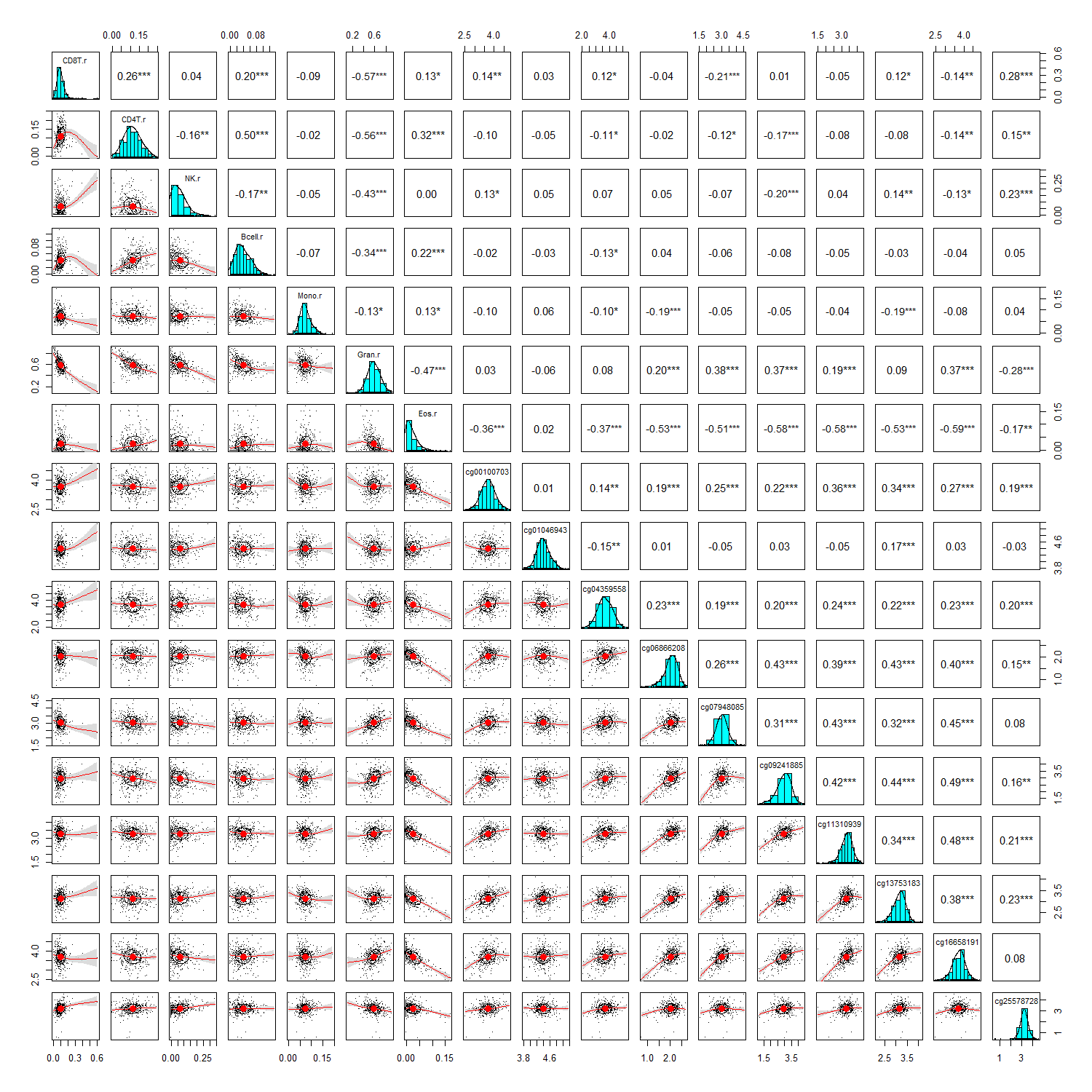

Supplement: Supplementary file 3 — Additional file 3: This file includes supplemental figures (S1, S2)—Figure S1. Tracking of the misclassification rates (y-axis) across iterations (x-axis) of the recursive RF feature selection. Figure S2. Correlations (Spearman) between estimated cell-proportions in blood and DNAM M-values for the 10 CpGs that were identified as candidates for the replication study. Statistically significant correlations are designated at p-values < 0.05 (*), p-values < 0.001 (**), and p-values < 0.001 (***). [file 13223_2019_356_MOESM3_ESM.docx]
